# MMR gene patterns evaluation provides novel insights for personalized immunotherapy compared to neoadjuvant chemotherapy in lung adenocarcinama

**DOI:** 10.1186/s12885-023-10905-3

**Published:** 2023-06-06

**Authors:** Liangliang Cai, Hujia Hua, Xingyu Jiang, Xintian Xu, Hua Bai, Li Qian, Jianchun Duan

**Affiliations:** 1 Institute of Translational Medicine, Medical College, Yangzhou University, Yangzhou, 225001 P.R. China; 2 Jiangsu Key Laboratory of Experimental & Translational Non-coding RNA Research, Yangzhou, 225001 P.R. China; 3 CAMS Key Laboratory of Translational Research on Lung Cancer, State Key Laboratory of Molecular Oncology, Department of Medical Oncology, National Clinical Research Center for Cancer/Cancer Hospital, National Cancer Center, Chinese Academy of Medical Sciences Peking Union Medical College, Beijing, 100021 China; 4 No. 48 East Wenhui Road, Yangzhou, Jiangsu 225009 China

**Keywords:** LUAD, MMR, Prognosis, TCGA-LUAD, Neoadjuvant Chemotherapy

## Abstract

**Background:**

The association involving mismatch repair (MMR) genes, molecular subtype and specific immune cell group in tumor microenvironment has been focused by more recent studies. Its prognosis value in lung adenocarcinoma (LUAD) neoadjuvant chemotherapy remains elusive.

**Methods:**

The correlation between the MMR gene patterns and the immune landscape were comprehensively evaluated. The MMRScore was calculated using principal component analysis (PCA) after grouping using R/mclust package. The prognostic significance of the MMRScore was evaluated by Kaplan-merrier analysis. Then a cohort of 103 Chinese LUAD patients was collected for neoadjuvant chemotherapy prognosis evaluation and validation using MMRScore.

**Results:**

Four MMRclusters (mc1, 2, 3, 4)-characterized by differences in extent of aneuploidy, expression of immunomodulatory (IM) genes, mRNA expression, lncRNA expression and prognosis were identified. We established MMRscore to quantify the MMR pattern of individual LUAD patients. As is shown in further analyses, the MMRscore was a potential independent prognostic factor of LUAD. Finally, the prognostic value of the MMRscore and its association with tumor immune microenvironment (TIME) of LUAD were verified in Chinese LUAD cohort.

**Conclusions:**

We demonstrated the correlation between MMR gene pattern, the CNV and tumor immune landscape in LUAD. A MMRcluster mc2 with high MMRscore, high TMB and high CNV subtype was identified with poor prognosis and infiltrating immunocyte. The comprehensive evaluation of MMR patterns in individual LUAD patients enhances the understanding of TIME and gives a new insight toward improved immune treatment strategies for LUAD patients compared to neoadjuvant chemotherapy.

## Background

DNA repair is necessary to ensure cellular genome integrity, served as a multi-enzyme, multi-pathway system [1]. DNA damage can occur either through errors during DNA replication or through chemical changes in base nucleotides in the cellular environment. Several mechanisms such as nucleotide excision repair (NER), base excision repair (BER), DNA strand break repair (DSBR), direct reversal of DNA damage, and replication of DNA damage by specialized DNA-derived polymerases (bypass replication), which are fundamental underlying mechanisms in different DNA repair pathways [2]. The occurrence of many specific human diseases is thought to be related to these defects in the repair pathways. In addition, previous studies have long found that the repair of damaged DNA is closely related to a variety of cellular processes such as DNA replication, DNA recombination, and cell cycle checkpoint arrest.

Mismatch repair (MMR) genes produced by DDR pathway is the most known type of DNA mutation cause [3]; it is although to regulate multiple DNA-related processes, such as DNA stability and alternative splicing [4]. Recently, aberrant expression of MMR regulators is revealed to be associated with cancer and immune events including tumorigenesis, immunomodulatory (IM) abnormality and malignant tumor progression [5].

Neoadjuvant chemotherapy is a treatment method in which some cycles of chemotherapy are given before tumor resection and the remaining cycles are given after surgery. Neoadjuvant chemotherapy is considered as a therapy with multiple potential benefits in the treatment of lung cancer [6]. For patients with one of these types of tumors that do not respond well to chemotherapy, the value of that treatment is diminished if therapeutic interventions to improve outcomes for such patients cannot be identified.

The association involving mismatch repair (MMR) genes, molecular subtype and specific immune cell group in tumor microenvironment has been focused by more recent studies. Its prognosis value in lung adenocarcinoma (LUAD) neoadjuvant chemotherapy remains elusive. In this investigation, we integrated the clinical and molecular data of 461 TCGA-LUAD and 103 Chinese LUAD cancer patients to comprehensively evaluate the MMR modification patterns and with lung cancer neoadjuvant chemotherapy. Distinct MMR modification regulation pattern and associated distinct immune characters, neoadjuvant chemotherapy sensitivity and prognoses were identified, showing the key roles of MMR gene pattern in the developments of individual TIME in lung adenocarcinoma patients. We also construct a methodology to quantify the MMR modification of individual LUAD patients by evaluating the gene patterns of 24 MMR regulators.

## Methods

### Molecular and clinical data

RNA sequencing data (count and fpkm values) for gene expression analysis, genetic mutations (Mutect2), and clinical data were downloaded from the Genomic Data Commons (https://portal.gdc.cancer.gov/). The Ensembl gene IDs of the RNA-seq data were mapped to gene symbols by referring to an annotation file (https://www.gencodegenes.Org/human/release_22. html). The copy number variation (CNV) data were downloaded from the xena web tool (https://xena.ucsc.edu/) [7].

### Model-based clustering analysis for MMR regulators

Gene expression levels were quantified using the metric log2 (fpkm + 1), then used to identify MMR modification patterns based on the expression of 24 MMR regulators genes by model-based clustering analysis implemented in the R package/mclust [8]. In this package, the optimal number of clusters was determined based on the Bayesian information criterion (BIC).

### Gene set variation analysis (GSVA)

Gene set variation analysis (GSVA)-a non-parametric and unsupervised method commonly used for estimating pathway variations in the samples of expression datasets-was performed to explore the differences in biological processes among MMR modification patterns [9]. The c2.cp.kegg. v6.2. symbols gene sets for GSVA were downloaded from the Molecular Signatures Database (MSigDB). A p < 0.05 was considered statistically significant.

### Identification of differentially expressed genes (DEGs) among MMRclusters

To identify genes related to MMR modification regulation, we classified LUAD patients into MMRclusters based on the expression of 24 MMR genes. DEGs among these clusters were determined using the R/limma package, which was applied using the raw fpkm values of RNA sequencing data. Genes with adjusted p < 0.05 and at least two-fold changes in expression were identified as DEGs. We refer to the research method of Zeng et al. [10].

### Construction of the MMR gene signature

In this part, we refer to the research method of Zeng et al[10]. We applied a methodology to quantify the MMR modification pattern (MMRscore) of individual LUAD patients. The MMRScore was established as follows. First, we extracted the overlapping DEGs among MMRClusters and classified the LUAD patients into several groups using model-based clustering to analyze the overlapping DEGs. Univariate Cox regression analysis was performed to evaluate the prognosis of each overlapping DEG. Genes with a significant prognosis (p < 0.05) were extracted for further analysis. Next, principal component analysis (PCA) was performed to establish the MMR gene signature. We selected both principal components 1 (PC1) and 2 (PC2) as signature scores. Finally, the MMRscore was defined using a method similar to Genomic Grade Index:

$$MMRScore{\text{ }} = \Sigma \left( {PC1i{\text{ }} + {\text{ }}PC2i} \right)$$

where i is the expression of overlapping genes with a significant prognosis of DEGs among MMRclusters.

### Correlation between MMRscore and other relevant biological processes

In this part, we refer to the research method of Zeng et al. [10] Spearman’s correlation analysis was performed to explore the correlation between MMRscore and other relevant biological processes using the gene sets reported by Mariathasan et al., including (1) antigen processing machinery (APM), (2) effector CD8 T-cell signature, (3) immune checkpoint, (4) nucleotide excision repair, (5) mismatch repair, (6) DNA replication, (7) DNA damage repair, (8) epithelial-mesenchymal transition markers, (9) Wnt targets, (10) pan-fibroblast transforming growth factor-β response signature, and (11) angiogenesis signature.

### Statistical analysis

Statistical significance for 3 or more groups was estimated using the Kruskal-Wallis test and association between categorical variables was explored using the χ2 test. The correlation coefficient was calculated via Spearman’s correlation analysis. The Kaplan-Meier method was used to generate survival curves and the log-rank test was used to determine the statistical significance of differences. The oncoplot function of R/maftools package was used to depict the mutation landscape of TCGA-LUAD cohort and immunotherapeutic cohort. All tests were two sided, and p < 0.05 was regarded as significant. All analyses were performed with R software V.4.2.0 (http://www.R-project.org).

## Results

### MMR regulators in LUAD: mutation landscape characteristics and clinical relevance

**Fig. 1:**
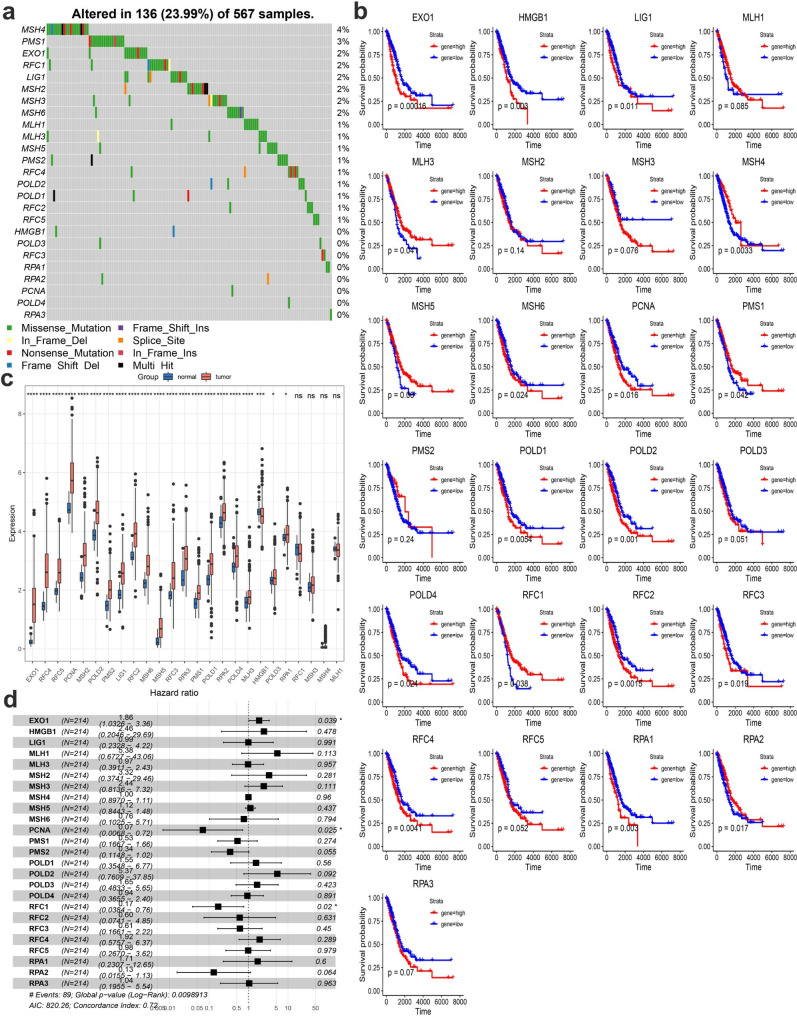
Clinical relevance and molecular characteristics of MMR regulator genes in LUAD. (**A**) The mutation landscape of 24 MMR regulator genes in 492 LUADs; (**B**) The overall survival of high or low expression of 24 MMR regulator genes in LUADs; (**C**) The gene expression alterations among MMR regulator genes; Tumor (normal) was indicated in red (blue). ANOVA test: The asterisks represented the statistical p value (*P < 0.05; **P < 0.01; ***P < 0.001); (**D**) For clinical relevance evaluation, a COX model analysis shows positive related genes

Based on published literature, DNA miss match repair (MMR) is regulated by 24 genes were highlighted. The mutation frequency of 24 MMR genes changes in LUAD was investigated using somatic mutations. Only 18 of 567 samples had MMR regulator mutations, indicating that a complete average mutation frequency of MMR regulators was lower (Please see in Fig. 1a). The survival curve of the 24 MMR regulators was then examined, and it was shown that 16/24 MMR regulators had a substantial influence (p < 0.05) on LUAD patients (Please see in Fig. 1b). The MMR regulators’ mRNA expression levels in LUAD and surrounding tissues were also investigated, and it was discovered that 22 of the 24 MMR regulators were differently expressed with p < 0.05 (Please see in Fig. 1c). The expressional and genetic differences in MMR regulators were significantly diverse between LUAD and surrounding tissues, indicating that MMR regulator expression imbalance plays a critical role in formation and progression of LUAD. For clinical effect evaluation, we execute a COX model which shows that HDAC1 has positive correlation (Please see in Fig. 1d). Taken together, these findings suggest that MMR gene pattern plays critical roles in the formation of LUAD.

### MMR gene patterns mediated by 24 MMR regulators

**Fig. 2:**
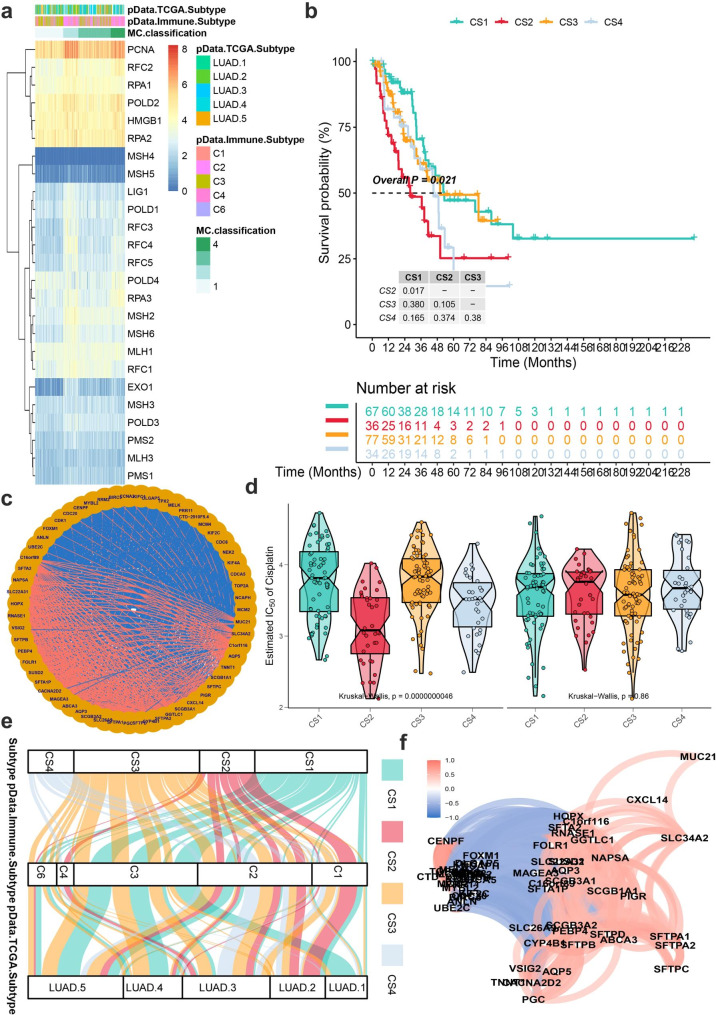
MMR modification patterns in LUAD and biological characteristics of MMR subtypes. (**A**) Model-based clustering of LUAD yields four subtypes in the TCGA-LUAD dataset. MC1, cluster1; MC2, cluster2; MC3, cluster3; MC4, cluster4; (**B**) Comparison of prognosis among four Chromatin Modification subtypes (Kaplan-Meier analysis); (**C**) PPI network based on 41 COX DEGs; (**D**) Boxviolins for estimated IC50 of Cisplatin and Paclitaxel among 5 identified subtypes in TCGA-LUAD cohort; (**E**) Agreement of 5 identified subtypes with immune status and pathological stage in TCGA-LUAD cohort; (**F**) PPI network based on 41 COX DEG using network

The 24 MMR regulators’ expression was used to categorize LUAD patients using model-based clustering. We found four different RNA methylation modification patterns (called MMRClusters MC1–MC4), with 118 cases in MMRcluster-C1, 129 cases in MMRCluster-C2, 53 cases in MMRCluster-C3, and 85 cases in MMRCluster-C4 (Please see in Fig. 2a). RFC4, MSH2, EXO1 and POLD3 expression levels were high in MMRCluster-MC2, whereas none was low. And MMRCluster-MC2 had the poor prognosis with p < 0.05 (Please see in Fig. 2b).

The R/limma package software was used to find 48 DEGs associated to the four-cluster subtype. The network activity of DEGs was investigated (Please see in Fig. 2c). Based on four MMRClusters, the therapy sensitivity of chemotherapy was evaluated (Please see in Fig. 2d), cisplatin shows lower IC50 than paxitiex. Study had investigated the pan-cancer immune landscape and eventually found the six immune subtypes (C1–C6) considered for determining the immune response patterns and have consequences for future immunotherapy research. In most LUAD patients, the immune subtype C3 was enriched, which is characterized by lower levels of overall CNVs. Surprisingly, the four unique MMR gene pattern levels showed different C3 immune subtype proportions, with MMRCluster-C3 in Fig. 2e. For more detailed description, we execute a DEGs relationship analysis as the same for network plot in Fig. 2f.

### Immune characteristics and subtype identification in distinct MMR modification patterns

**Fig. 3:**
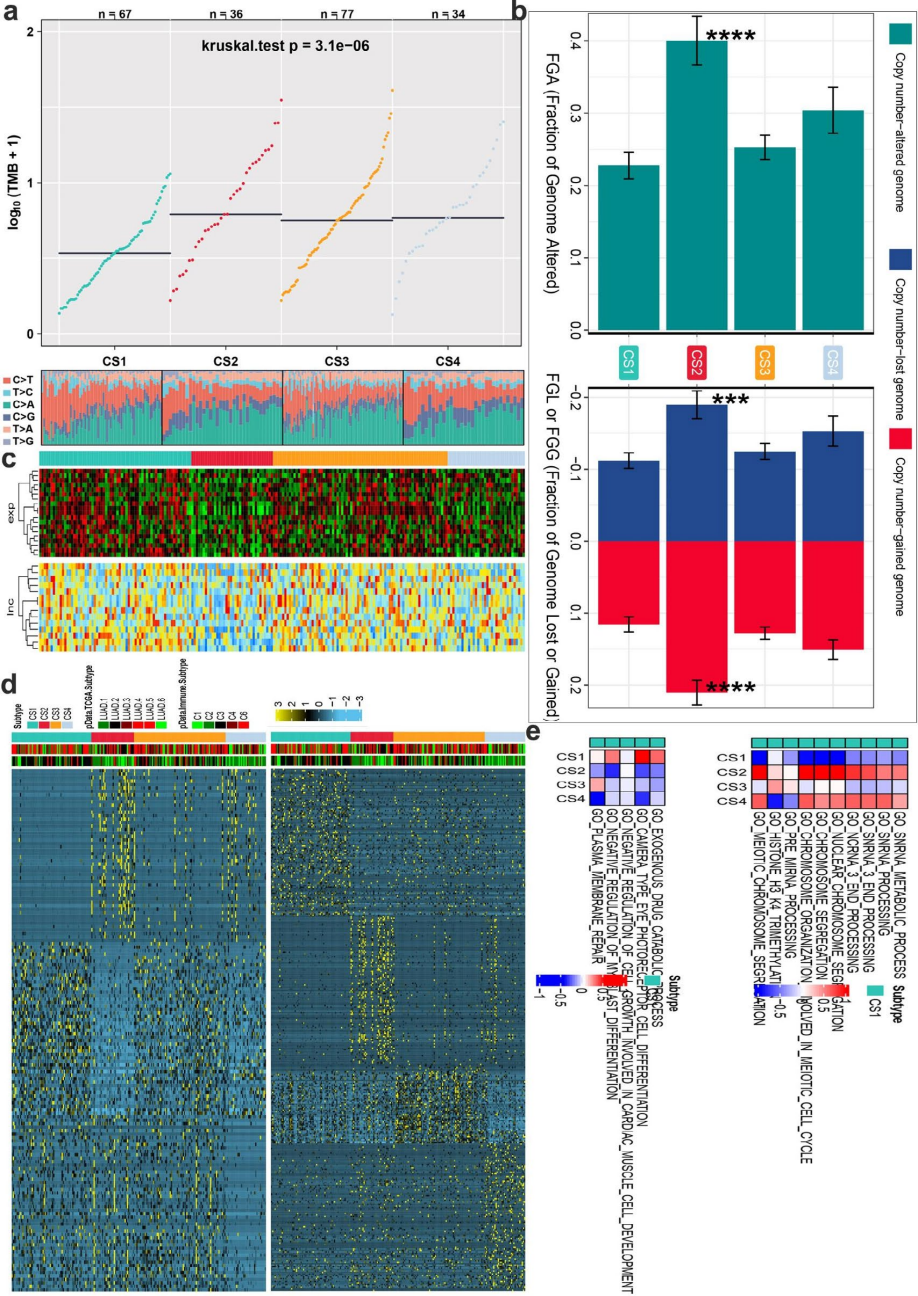
Different responses of immune cells are enriched in the four subtypes of lung cancer. (**A**) Comparison of TMB and TiTv among four identified subtypes of lung cancer in TCGA-LUAD cohort; (**B**) Barplot of fraction genome altered among four identified subtypes of lung cancer in TCGA-LUAD cohort; (**C**) Molecular subtypes in distinct MMRclusters. From top to bottom: mRNA expression (median normalized expression levels); lncRNA expression (median normalized expression levels); (**D**) Heatmap of subtype-specific upregulated and downregulated biomarkers using limma for 4 identified subtypes in TCGA-LUAD cohort; (**E**) GSVA of subtype-specific upregulated pathways (left). GSVA of subtype-specific downregulated pathways in TCGA-LUAD cohort (right)

Thorsson et [11] colleagues investigated the pan-cancer immune landscape and eventually found the six immune subtypes (C1–C6) considered for determining the immune response patterns and have consequences for future immunotherapy research. Surprisingly, among the four MMRclusters, the 4 unique modification levels showed different C1 and C2 immune subtype proportions, with MMRcluster-C2 having highest, followed by MMRcluster-C3, and C1 (p < 0.001). The immunological properties of various MMR patterns were next investigated in further detail. In comparison to the other clusters, MMRcluster-C2 had a high TMB, higher levels of overall CNVs and different mRNA and LncRNA expression pattern (Please see in Fig. 3a-c). Subtype-specific upregulate or downregulate biomarkers were found by starting with differential expression analysis (DEA). The most differentially expressed genes (DEG) sorted by log2Fold are chosen as the biomarkers for each MMRCluster subtype. These biomarkers should pass the R/limma package analysis to identify subtype-specific downregulated Fig. 3d in left and upregulated in right biomarkers.

Similarly, GSEA is executed for each molecular subtype based on the DEA results to identify signal pathways using a gene set background which includes all gene sets derived from GO biological processes (c5. bp.v 7.1. symbols. gmt). Heatmap analysis of subtype specific downregulated biological pathways (Please see in Fig. 3e top) using limma package for 6 identified subtypes in TCGA-LUAD and upregulated pathways (Please see in Fig. 3e bottom).

### Construction of the MMR gene signature and evaluated the immune landscape was significantly associated with MMRScore

**Fig. 4:**
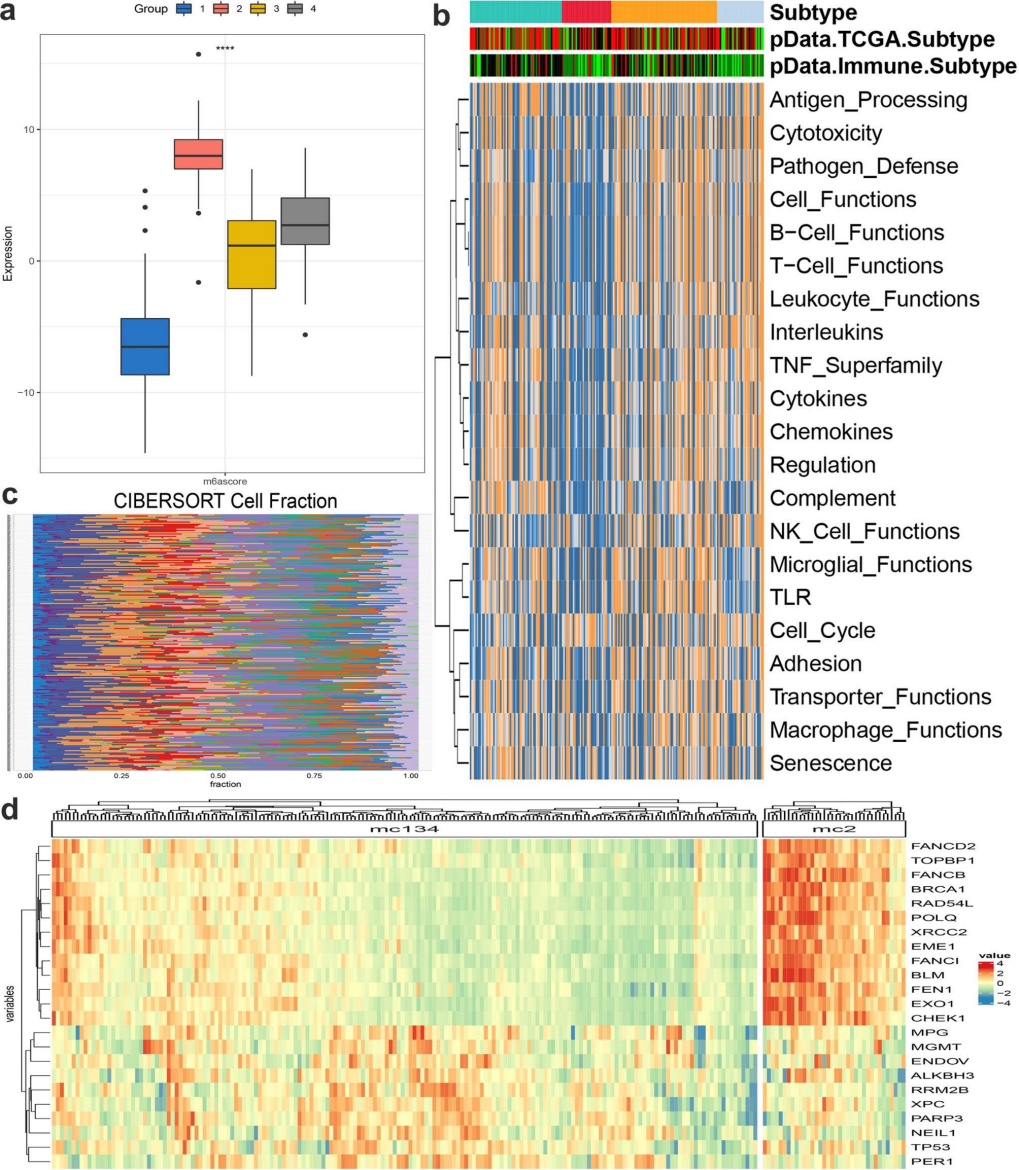
The immune landscape in distinct MMR modification patterns. (**A**) Box plot visualizing the chromatin modification of four clusters. Boxplot showing the different chromatinScore between chromatin modification subtypes. ANOVA test: The asterisks represented the statistical p value (*p < 0.05; **p < 0.01; ***p < 0.001); (**B**) Heatmap of enrichment score of gene set of interest for four identified subtypes in PRAD; (**C**) The distribution of 22 types of immune cells between normal and tumor of PRAD cancer using CIBERSORT; (**D**) Heatmap plot showing the different immune related genes between chromatin modification subtypes

The immunological properties were next further explored in MMR gene patterns. 49 DEGs associated with significant prognoses were extracted for further PCA analysis to establish the chromatin modification gene signature. Based on the visualized box plot in Fig. 4a, we could find that a positive differentiation (p < 0.05) between the four MMRClusters. Furthermore, the student t test showed a positive difference in MMRScore among all the four MMRClusters. We used the MMRScore assay to assess the MMR alteration pattern in individual LUAD. The R/limma program software was used to find 49 DEGs associated to the mc2 subtype. The prognosis of 49 genes in the MMR modification subtype associated DEGs were assessed using a univariate Cox regression analysis.

Exploring the expression of diverse MMR alteration patterns is required to progress this study. The functions based on the expression of IM regulators in the MMR 4 subtypes were investigated in Fig. 4b. Almost all function were poorly expressed in mc2 especially in immune, such as T function, B function, APC processing, Macrophage functions. As shown in Fig. 4c, using the CIBERSORT algorithm, it is not difficult to find B cell memory, B cell naive, macrophages M0, M1, T cell CD4 memory and the dendritic cells showed an activated state. In particular, quiescent dendritic cells showed significant differences between normal and cancerous tissues, respectively. The heatmap of immune related genes between mc1 and mc134 lung adenocarcinoma samples was shown in Fig. 4d.

### MMR-associated immune microenvironment characteristics in validation cohort

**Fig. 5:**
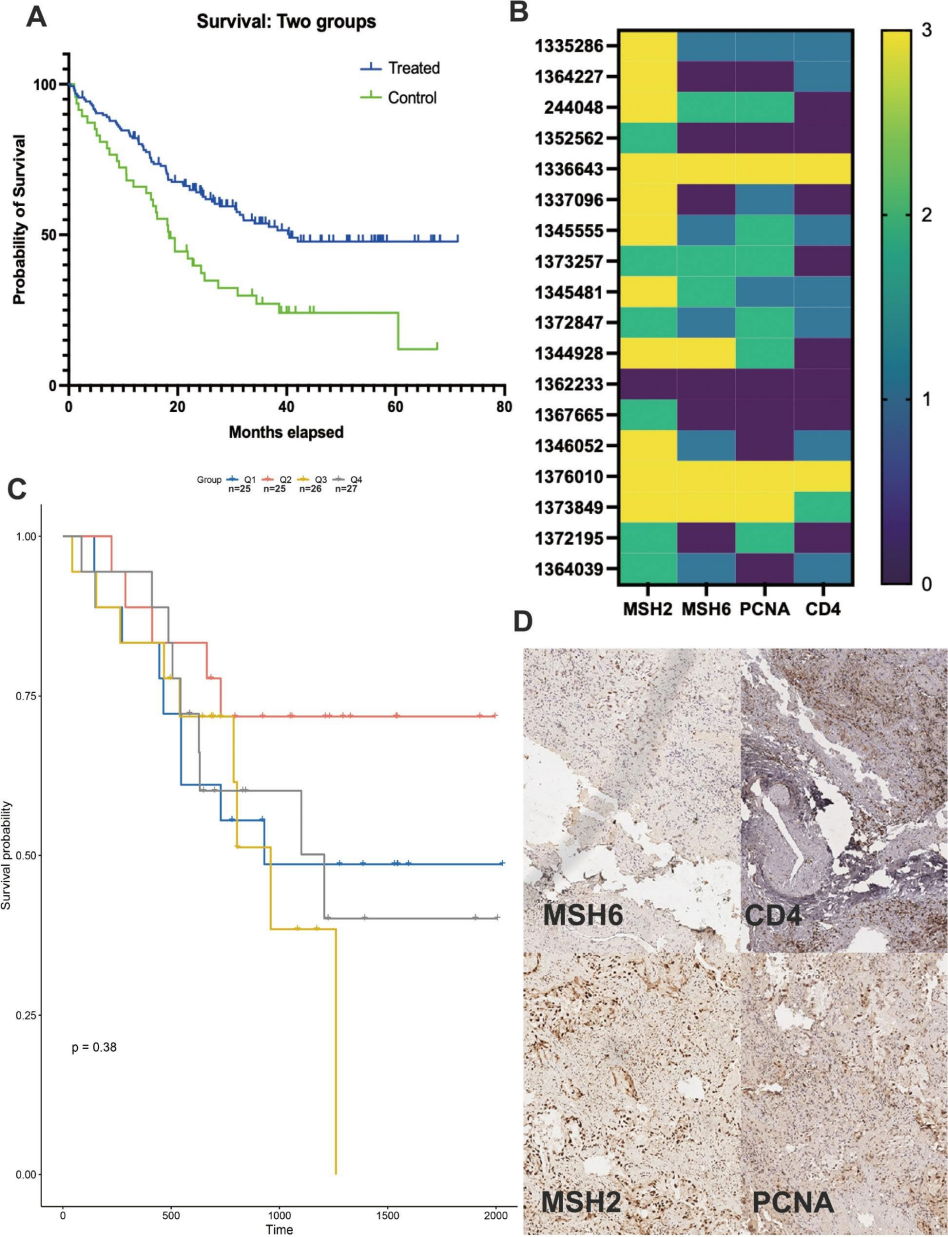
MMR-Associated Immune Microenvironment Characteristics in validation cohort. (**A**) Kaplan-merie analysis of collected 102 Chinese LUAD patients; (**B**) Heatmap plot of IHC detection of MSH6, MSH2 and CD4 in 8 sample; (**C**) Kaplan-merie analysis of four subtypes according to MMRScore; (**D**) The IHC image of one sample of MSH6, MSH2 and CD4.

For further validation our previous results, we collect a Chinese LUAD cohort including 103 Chinese LUAD patients with DFS after neoadjuvant chemotherapy. With or no neoadjuvant chemotherapy, Kaplan-merrier plot analysis in Fig. 5a shows a significant effect after neoadjuvant chemotherapy. With a consideration of subtyping, we recalculated the MMRScore for every individual in the 103 Chinese cohort. Using median method, the cohort was for Kaplan-merrier plot analysis in Fig. 5b. Antigen processing, chemokines, and responses to interferons were all significantly up-regulated in the high MMRScore group, suggesting an increase in the efficiency of T cell recognition of antigens and thereby triggering inflammation and antitumor immunity. We then assessed the protein expression of PD-L1 and CD4 in primary lung cancer based on the four MMRclusters in Fig. 5c using immunohistochemical (IHC) analysis, and some representative pictures in Fig. 5d. In the subtype of validation cohort, mc3 shows a higher mRNA expression gene a, lower gene b and dysfunction immune. For further validation, we detect their expression using IHC in 4 Chinese LUAD cohort with higher MMRScore, the data shows that higher mRNA expression gene a, lower gene b was associated with low CD4 expression.

### Functional annotations and pathway enrichment analyses of MMRScore subtypes

**Fig. 6:**
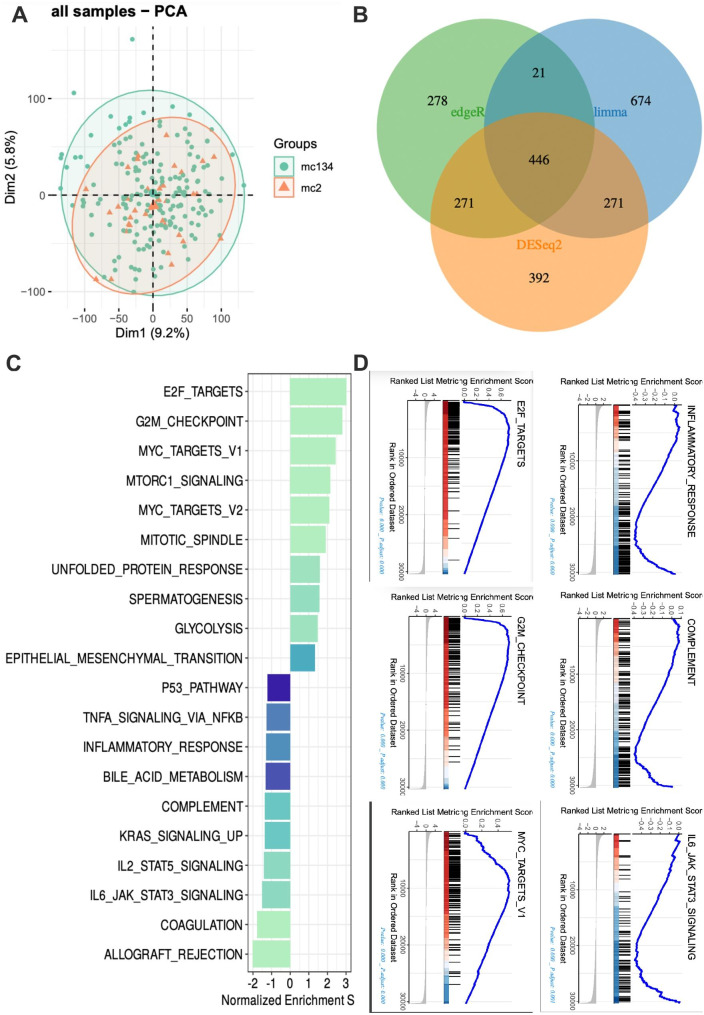
Functional Annotations and Pathway Enrichment Analyses of MMRScore Subtypes. (**A**, **B**) RNASeqstat2 pipeline: PCA analysis and venn plot of three methodologies (limma, edgeR and DEseq2). (**C**) GSEA HALLMARK analysis of the mc2 and mc134 MMRscore groups. (**D**) HALLMARK enrichment of mc2 and mc134 subtypes calculated by the ssGSEA algorithm with activation or inhibition

To understand the MMRscore’s potential biological power in terms of remarkable predictive ability, we analyzed the GO enrichment in TCGA with the help of the R/clusterProfiler package, where the analyzed data came from PCA and DEG of the RNASeqstat2 pipeline (Please see in Fig. 6a and b). KEGG and GSEA analysis showed that, among all pathways, synaptic transmission pathways were enriched in the low MMRscore group, and pathways involved in immune system diseases were significantly associated with high MMRscore in Fig. 6c. On this basis, for the HALLMARK and KEGG signature scores of each sample, we use the ssGSEA algorithm to conduct a comprehensive exploration. Adjusted p-values were ranked by the limma algorithm according to their significance, and the top 20 enrichments were visualized in a heatmap (Please see in Fig. 6d). High MMRscore LUAD showed enrichment of multiple immune activation pathways including IL-6/JAK/STAT3 pathway, interferon-gamma response, inflammatory response, and antigen processing and presentation. Low MMRscore is related to the enrichment of E2F_TARGETS and G2M_checkpoint.

## Discussion

A growing number of studies have shown that MMR genes play critical roles in tumorigenesis, therapeutic clinical resistance, and immune responses through the cooperation between MMR regulators [4, 5]. At present, questions about the role of MMR modification patterns in the tumor immune microenvironment have been explored and answered in some cancer types. In this investigation, we further deepen our understanding of TIME-based antitumor immune responses by exploring and understanding the role of MMR gene patterns in the immune landscape of lung adenocarcinoma and use this as a springboard to provide more effective LUAD patients. immunotherapy strategies.

Lung cancer can already be subtyped based on genomic profiling, which promises to improve the application of precision-focused personalized therapy in the future [12]. In this study, among 24 MMR regulators, four MMR-related clusters with significantly different immune microenvironments have been specified, based on their differences in aneuploidy, overall somatic copy number alterations, expression of immune-related genes and prognosis. In addition, Th17 expression is often associated with improved prognosis in previous studies [13]. Coincidentally, this experiment also draws consistent conclusions as follows: C3 presents enriched pathways associated with complete immune activation and exhibits the most obvious Th17 signature. MMRcluster-C1 exhibited not only high proliferation and ITH, but also enriched pathways associated with full immune activation and relatively high CD8 + T cell infiltration, all of which indicate high tumor growth rates in C1. So, it may seem strange but not contradictory that C1 shows a state of activated immunity but at the same time there is a low survival rate. MMRcluster-C2 exhibits features that are primarily associated with immunosuppression of biological processes and relatively low infiltration of CD8 + T cells.

Because each individual has a different MMR modification pattern, we adopted a methodology called MMRscore to accurately calculate the MMR pattern of different lung adenocarcinoma patients. Our study also found that MMRScore was positively correlated with CNV. Through comprehensive analysis, we identified MMRScore as a potential independent prognostic factor for neoadjuvant chemotherapy. Therefore, we judged that the MMR gene pattern may serve as a key influencer leading to different clinical responses to immunotherapy and indirectly validated the value of MMRscore in predicting immunotherapy outcome.

The advent of anti-PD-1/PD-L1 ICT therapy is a breakthrough in the treatment of certain advanced cancer types [14, 15]. However, LUAD patients who received this treatment did not all have a positive and significant clinical response, and immunotherapy results showed individual heterogeneity. Therefore, it is very important to find markers that can predict the outcome of immunotherapy. It is known that if people have pre-existing CD8 + T cell infiltration and high tumor mutational burden, they can have a higher response to anti-PD-1 therapy. However, in some advanced cancers, contrary to some known cancer types, tumor mutational burden, neoantigen burden, and HLA engagement did not correlate with response to anti-PD-1 therapy. And in other cancer type, immune-infiltrating tumors and immune-desert/excluded tumors, respectively, did not differ statistically in response or survival to anti-PD-1 therapy. In our study, we discovered and confirmed the prognostic value of MMRscore in cold-immunized LUAD patients with low T cell infiltration and used MMRscore as a predictive strategy for anti-PD-1/PD-L1 therapy.

Neoadjuvant chemotherapy can theoretically improve disease-free survival (DFS) and overall survival (OS) by early treatment of microscopic metastases. From a practical standpoint, neoadjuvant chemotherapy provides sufficient time for other surgical planning such as custom endoprosthesis preparation. In addition, neoadjuvant chemotherapy can make patients who would otherwise be inoperable and must have their limbs amputated eligible for surgery if they shrink the tumor sufficiently [16]. At the same time neoadjuvant chemotherapy can improve postoperative healing because recovery from chemotherapy is less time-critical. One of the most important ways to improve prognosis is early identification combined with subsequent treatment. However, there are currently no known therapeutic interventions to improve outcomes for patients with poor histologic response to chemotherapy [17]. Therefore, we believe that MMRscore has adjuvant predictive value for neoadjuvant chemotherapy.

Finally, this investigation discovered a link between MMR gene pattern, copy number variation (CNV), and the immunological landscape of lung cancer tumors. With a clinical cohort to verify our pre results on TCGA-LUAD, our in-depth analysis of MMR alteration patterns in individual lung cancer patients adds to knowledge of tumor immunological landscape and paves the way for neoadjuvant chemotherapy prognosis or novel and better immunotherapeutic methods. In our study, we have a disadvantage due to the lack of enough clinical data on immune checkpoint therapy. This is an important factor to consider, as it can limit the validity and applicability of the research findings.

## Data Availability

8 humans are involved in the study for IHC assay. All data used in this work can be acquired from the GDC portal (https://portal.gdc.cancer.gov/), and the website (https://gdc.cancer.gov/ about- data/ publications/ panimmune).
